# Implementation of a Community-Based Mind–Body (Tae-Bo) Physical Activity Programme on Health-Related Physical Fitness in Rural Black Overweight and Obese Women with Manifest Risk Factors for Multimorbidity

**DOI:** 10.3390/ijerph20156463

**Published:** 2023-07-27

**Authors:** Musa Mathunjwa, Ina Shaw, Jason Moran, Gavin R. Sandercock, Gregory A. Brown, Brandon S. Shaw

**Affiliations:** 1 Department of Human Movement Science, University of Zululand, KwaDlangezwa 3886, South Africa; mathunjwam@unizulu.ac.za; 2 School of Sport, Rehabilitation and Exercise Sciences, University of Essex, Wivenhoe Park, Colchester CO4 3SQ, UK; i.shaw@essex.ac.uk (I.S.); jmorana@essex.ac.uk (J.M.); gavins@essex.ac.uk (G.R.S.); 3 Physical Activity and Wellness Laboratory, Department of Kinesiology and Sports Science, University of Nebraska Kearney, Kearney, NE 68849, USA; brownga@unk.edu

**Keywords:** exercise, intervention study, obesity, obesity management, overweight

## Abstract

Non-communicable diseases (NCDs) are the leading cause of death globally, particularly impacting low- and middle-income countries and rural dwellers. Therefore, this programme aimed to investigate if a community-based mind–body PA programme implemented in a low-resource setting could improve health-related physical fitness outcomes. Black overweight or obese adult women (25 ± 4.7 years) with a body mass index (BMI) > 25 kg·m^−2^ recruited from a rural settlement in South Africa with manifest risk factors for multimorbidity were assigned to a 10-week waiting-to-treat non-exercising control group (*n* = 65) or a community-based mind–body programme (*n* = 60) consisting of 45–60 min, thrice-weekly Tae-Bo. The intervention resulted in significant (*p* ≤ 0.05) improvements in body weight (*p* = 0.043), BMI (*p* = 0.037), and waist (*p* = 0.031) and hip circumferences (*p* = 0.040). Flexibility was found to be significantly increased at mid- and post-programme (*p* = 0.033 and *p* = 0.025, respectively) as was static balance (mid: *p* = 0.022; post: *p* = 0.019), hand grip strength (mid: *p* = 0.034; post: *p* = 0.029), sit-up performance (mid: *p* = 0.021; post: *p* = 0.018), and cardiorespiratory endurance (mid: *p* = 0.017; post: *p* = 0.011). No significant change was found in sum of skinfolds following the programme (*p* = 0.057). Such a community-based mind–body programme presents an opportunity to level health inequalities and positively improve health-related physical fitness in low-resource communities irrespective of the underlying barriers to participation.

## 1. Introduction

South Africa, and Africa in general, is experiencing a challenge of dealing with multiple burdens of disease arising from both infectious diseases, such as human immunodeficiency virus (HIV), acquired immunodeficiency syndrome (AIDS), and tuberculosis (TB), and non-communicable diseases (NCDs), such as cardiovascular diseases and diabetes [1,2,3]. Of these diseases, NCDs are the leading cause of death globally with approximately 75% of NCD-related deaths occurring in low- and middle-income countries (LMICs) [4]. Non-communicable diseases (NCDs), or lifestyle diseases, are primarily due to tobacco use, unhealthy dietary patterns, alcohol abuse, and lack of regular physical activity [5].

While country-wide policies exist in South Africa, and other African countries, for NCD risk factor and NCD intervention for tobacco use, alcohol abuse, and unhealthy dietary patterns, such policies are limited for physical inactivity [6]. In addition, when such policies do exist, they are rarely implemented optimally, if at all, due to numerous challenges, such as inadequate resources and skills and a lack of political intercession [7].

Physical activity has a large public health impact as it is associated with lower risks of NCDs and all-cause mortality in both males and females. Numerous studies have shown that exercise can improve several risk factors for many NCDs, such as decreasing blood pressure, improving lipoprotein lipid profiles, body composition, and cardiorespiratory endurance [8,9]. What is particularly interesting is that many of these benefits of regular physical activity are forthcoming even in the absence of weight loss [8]. While there is evidence for the benefits of physical activity intervention programmes on various NCDs in clinical settings [9] and/or high-resource settings [10], it is not evident that these evidence-based programmes can be implemented in a low-resource setting, such as rural or informal settings [11,12], or even if the same outcomes can be achieved in such settings [6]. As indicated by the World Health Organization (WHO), this lack of evidence has resulted in a lack of physical activity intervention guidelines being developed in LMICs [13], especially those that focus on rural communities at risk of or manifest with one or multiple NCDs. Further, when considering the increasing burden of NCDs in low-resource settings, the small body of evidence for physical activity interventions in sub-Saharan Africa and South America is particularly concerning [6]. Such evidence, when forthcoming, could inform the planning of future randomised controls (RCTs) and implementation science interventions in this context. This is because reporting on health-related outcomes that measure NCD risk or manifest risk could highlight the potential value of community-based physical activity programmes as an intervention.

Community-based physical activity programmes delivered in group settings could be an ideal vehicle to address NCD risk or manifest risk in isolation or in combination. This is because group-based physical activity programmes have previously been shown to address both physical and psychosocial aspects related to NCD risk while promoting social interaction, reducing drop-out rates, as well as generating a positive mind-set towards physical activity and a feeling of belonging, effectively improving long-term adherence [14]. However, when delivered in low-resource settings, such group intervention programmes should also be cognisant of the issues of cost effectiveness, resource requirements, transferability, and scalability of interventions to be delivered [12].

Previous studies utilising group-based mind–body physical activities, such as Tai-Chi, have demonstrated their ability to improve both mental and physical health [15]. Mind–body physical activity is a subdiscipline of movement therapy that refers to a broad range of Eastern and Western mindful movement-based practices, such as Tai-Chi, yoga, and Pilates, used to treat the mind and body simultaneously. However, many mind–body physical activity systems rely on coordinated slow, sedate, and meditational body-posture movements that may not suit all participants due to their complexity and sedateness. This has led to the development and investigation of more vigorous mindful movement-based practices, such as dancing, swimming, and Tae-Bo. In this regard, Tae-Bo, or Total Awareness Excellence Body Obedience, is a system that combines self-awareness and control using the movements of taekwondo, karate, boxing, and hip-hop dancing and is specifically designed to improve cardiorespiratory fitness, balance, and flexibility [16,17,18]. It is this combination of self-defence and dancing that may make this physical activity programme particularly appealing to rural community-dwellers living in rural and/or informal settlements. In addition, Tae-Bo is considered as a moderate-intensity physical activity [17], thus meeting international guidelines requiring moderate-intensity exercise to be performed for 150 min weekly [19]. As such, the aim of this programme was to investigate if a community-based mind–body physical activity programme could be implemented in a low-resource setting and if such a programme results in positive outcomes in health-related physical fitness in rural black overweight or obese women with manifest risk factors for multimorbidity.

## 2. Materials and Methods

### 2.1. Study Design and Setting

This study made use of a quasi-experimental, programme monitoring, and quantitative data evaluation in the rural Kwadlangezwa settlement in South Africa. This approach ensured that the participants served as their own control and that the intervention group had a stable baseline prior to engagement in the intervention, limiting the effect of confounding variables. This approach was also selected as it allowed all participants to undergo treatment simultaneously [20]. The Institutional Review Boards at the University of Zululand, South Africa provided ethical approval for this programme (UZREC 171110-030 PGM 2011/25). The programme complied with the tenets of the Declaration of Helsinki as revised in 2013.

### 2.2. Participants

One hundred and thirty-two overweight (body mass index (BMI) 25–29.9 kg per square m (kg·m^−2^)) or obese (BMI 30–39.9 kg·m^−2^) adult women (>18 years) with manifest risk factors for multimorbidity volunteered and were recruited using posters and flyers distributed with relevant permissions (i.e., traditional leaders) from the rural Kwadlangezwa settlement in KwaZulu-Natal, South Africa. Although no official statistics exist for the village of Kwadlangezwa, it has the following population demographics: 87.7% of the individuals in the municipality are African black, 7.3% are African white, with the other population groups making up the rest. Baseline characteristics are included in Table 1. Participants were assigned to a community-based mind–body physical activity programme (*n* = 67) or a waiting-to-treat non-exercising control group (*n* = 65). Eligibility criteria for inclusion in this programme required participants to have two NCD risk factors, namely overweight or obesity, and physical inactivity for at least six months prior to the programme [21]. Exclusion criteria included that all participants were to be free from any relative and absolute contraindications to exercise and exercise testing [18]. Further, a female-only sample was utilised to eliminate physiological and hormonal influence which may compromise the findings of the programme [22,23].

This programme followed a sequential style to implementation whereby clusters of participants began the programme in multiple phases following appropriately sensitive and cost-effective screening assessing the health-related physical fitness parameters of cardiorespiratory endurance, muscular fitness (including muscular endurance and muscular strength), body composition, flexibility, and balance [24]. Re-assessment of these parameters took place mid-programme and at completion of the programme. The same host administered each assessment, supervising the correct execution. In addition, adherence to the programme was recorded with records kept at every session of participation. Adherence was calculated as (sessions completed/total sessions expected) × 100. Further, after each session and at the completion of the programme, the hosts recorded any adverse events due to the intervention.

Kinanthropometric evaluations were completed as per the International Society for the Advancement of Kinanthropometry (ISAK) guidelines [25]. Each participant’s body weight (Growth Management scale, Genifis, China) and height (Seca Stadiometer 216, Seca, Hammer Steindamm, Germany) were evaluated. Body mass index (BMI) was determined with the following formula: BMI = BM/stature^2^. Waist-to-hip ratio (WHR) was calculated using waist (WC) and hip (HC) circumferences; WHR = WC/HC. Sum of skinfolds (ƩSKF) was determined using a Lange calliper (Rosscraft, Surrey, Canada) using the following skinfold measurements; triceps, subscapula, suprailiac crest, abdomen, thigh, and calf.

This programme made use of the sit-and-reach test (Podium 4 Sport Ltd., Belfast, UK) to measure flexibility since the process of performing this test is simplistic, requires minimum skills to administer, and is cost-effective and thus appropriate in a rural community with a large population size [26]. Participants were seated on the floor with both knees fully extended, shoulder width apart, and feet affixed in a dorsiflexion or plantar flexion ankle position. With one hand on top of the other, each participant exhaled and slowly reached forward as far as possible, holding that position for three seconds. Three trials of each test were performed following a five-minute rest interval with the highest score being recorded [27].

In this programme, the standing stork test was utilised to measure balance. Each participant was required to stand with their backs to the examiner. The examiner then placed their left thumb on the posterior superior iliac spinae (PSIS) with their right thumb palpating the midline of the sacrum at the same level as the PSIS. Each participant was asked to flex the left hip to 90° and maintain that position. The maximum time that each participant maintained the correct position was recorded [28].

Hand grip strength has increasingly been demonstrated as an indicator of physical health [29]. It is for this reason that the current programme utilised a hand grip test using a handheld dynamometer (Jamar digital dynamometer, Lancashire, UK). Participants stood upright with shoulders at 0° adduction and neutral rotation, elbows at 90° flexion, and forearms in the neutral position. Three tests for each arm were performed with a rest period of one minute between trials. Maximum values were recorded [30].

For the sit-up test, each participant was required to lie supine on the floor with 90° flexion in the knee joints, hands at the side of their head, and with elbows pointing straight forward. Each participant was required to flex their trunk until their elbows touched their knees followed by a return to the floor until their shoulders touched the floor. The number of correct repetitions performed in one minute was recorded [31].

Due to the nature of the participants and need for tests with ecological validity in low-resource settings, this programme made use of the YMCA submaximal three-minute step test. Participants stepped up and down a single 30 cm step set at a rate of 30 steps per minute for three minutes, in time with a Willner metronome (Malzel system, West Germany) [32].

The intervention required participants to engage in 10 weeks thrice-weekly group-led Tae-Bo sessions. We chose Tae-Bo since it aligns with both the affective–reflective theory (ART) of physical inactivity and exercise and the theory of energetic cost minimisation (TECM). These both address consideration-situated restraining forces, albeit in different ways. The ART is a psychological theory that relates individuals’ acute affective responses to exercise and how such experiences can influence the odds of future exercise. TECM has its roots in evolutionary behavioural biology and posits an ever-present restraining force in human behaviour toward efficiency in anticipation of potentially exhausting PA. Considering these two theories, the proposed intervention attempted to change individuals’ automatic reactions to PA-related stimuli and reduce the restraining forces. Once initiated, thrice-weekly sessions of mind–body Tae-Bo began. Each session consisted of 45–60 min of practical delivery. Sessions took place in indoor settings (e.g., community halls). The mind–body sessions were led by a qualified host and utilised a previously published protocol using 18–22 Tae-Bo exercises per session [17]. Sessions consisted of warm-up exercises comprising walking/jogging, step touch, double step touch, leg curl, double leg curl, knee up, and double knee up for 5–10 min. This was followed by the main workout, which lasted for 40 min, and a cool-down routine lasting 5–10 min consisting of two sets of eight whole-body static stretches held for 15–20 s. Prior to the intervention, participants were trained on the use of the Borg scale and were instructed to train at an intensity of 11–13 for the first five weeks and a rating of perceived exertion (RPE) of 14–16 for the final three weeks [17]. Training intensity in RPE was also recorded at each session. The waiting-to-treat control group were required to maintain their usual activities.

Standard statistical methods were used for the calculation of the means, standard deviations (SDs) and confidence intervals. To illustrate relative changes across the programme, percentage change (∆%) was calculated. A repeated measures analysis of variance (ANOVA) and a Tukey post hoc test were utilised to examine the differences between the baseline assessments and mid- and post-intervention. Effect size was calculated using the statistical calculation of Cohen’s d, and the standardised effect sizes were classified as small (<0.20), moderate (0.20–0.79), and large (>0.80). The rank-order procedure was utilised to rank the changes, or lack thereof, of each health-related physical fitness variable from 1 (for the smallest or most unfavourable change) to 10 (for the greatest or most favourable change). Correlations between the various dependent manifest risk factors, namely body composition (using BMI as a proxy) and physical fitness, were assessed with the other health-related physical fitness parameters using Spearman’s rank correlation coefficient. Data were analysed using version 25.0 of the IBM Statistical Package for the Social Sciences (SPSS) for Windows (IBM Corporation, Armonk, NY, USA).

## 3. Results

### 3.1. Adherence, Compliance, and Adverse Events

Of the 67 black overweight or obese adult women (age: 25 ± 4.7 years) that were recruited for the community-based mind–body physical activity programme, 60 completed the 10-week programme and were included in the final analysis. This represents an 89.6% adherence rate for this community-based mind–body physical activity programme. Reasons for this drop-out included: five women dropped out during the intervention phase due to personal reasons, and two women dropped out due to health reasons not related to the study. Further, participants included in the data analysis completed a mean of 27 (range 24 to 30) of a possible 30 sessions (96.7% compliance). Participants receiving the intervention were asked before and following sessions if they experienced falls, injuries, new muscle soreness, or pain because of the intervention exercises. Participants in the waiting-to-treat group were not queried about adverse events.

### 3.2. Outcome Measures

Across the 10-week waiting-to-treat non-exercising control period, no significant (*p* > 0.05) changes were found in any of the measured physical fitness parameters. Further, no significant differences were found between the two groups at baseline. The results for the effects of the community-based mind–body physical activity programme on health-related physical fitness are presented in Table 1.

**Figure 1:**
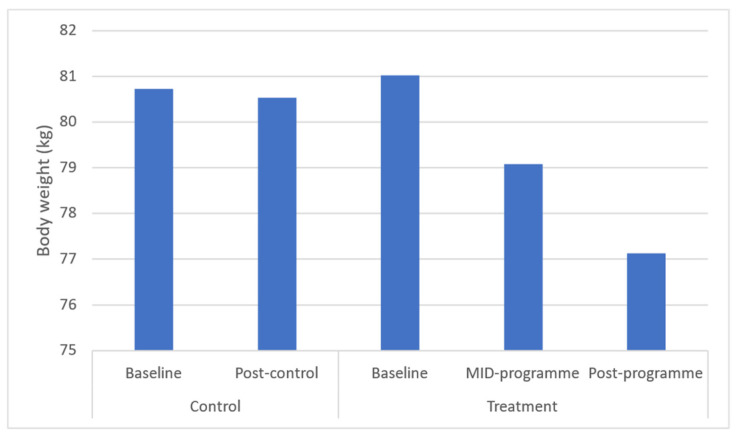
Effects of a community-based mind–body (Tae-Bo) physical exercise programme on body weight in rural black overweight and obese women with manifest risk factors for multimorbidity. Legend: kg: kilograms.

**Figure 2:**
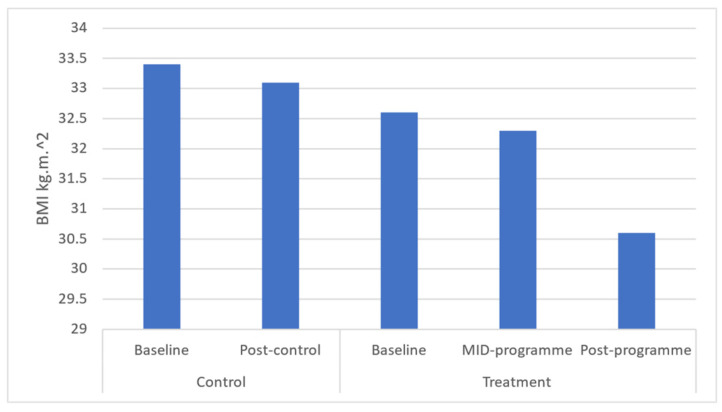
Effects of a community-based mind–body (Tae-Bo) physical exercise programme on body mass index (BMI) in rural black overweight and obese women with manifest risk factors for multimorbidity. Legend: BMI: body mass index; kg·m^−2^: kilograms per square metre.

**Figure 3:**
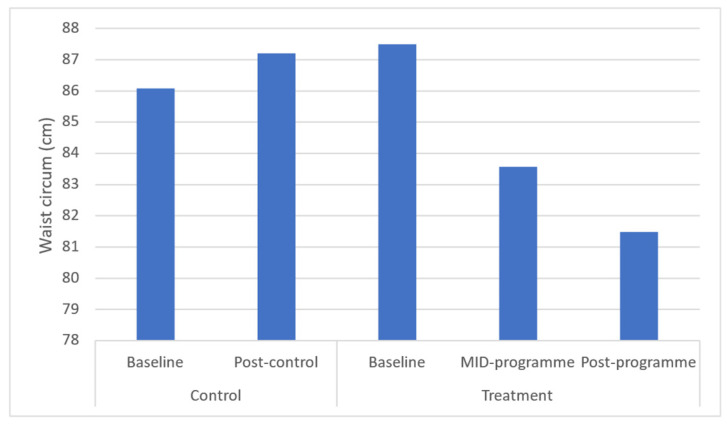
Effects of a community-based mind–body (Tae-Bo) physical exercise programme on waist circumference in rural black overweight and obese women with manifest risk factors for multimorbidity. Legend: waist circum: waist circumference; cm: centimetres.

**Figure 4:**
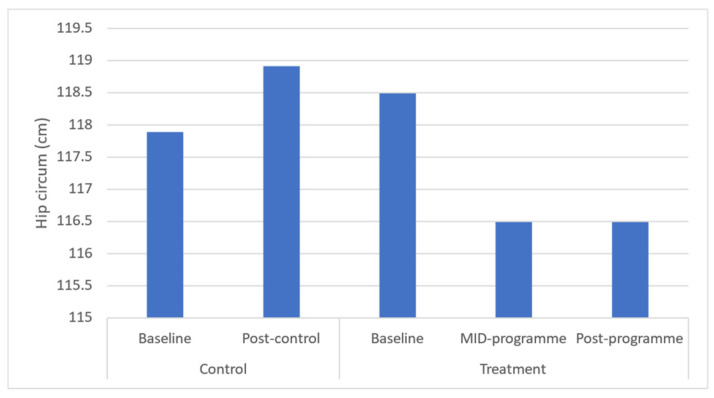
Effects of a community-based mind–body (Tae-Bo) physical exercise programme on hip circumference in rural black overweight and obese women with manifest risk factors for multimorbidity. Legend: hip circum: hip circumference; cm: centimetres.

**Figure 5:**
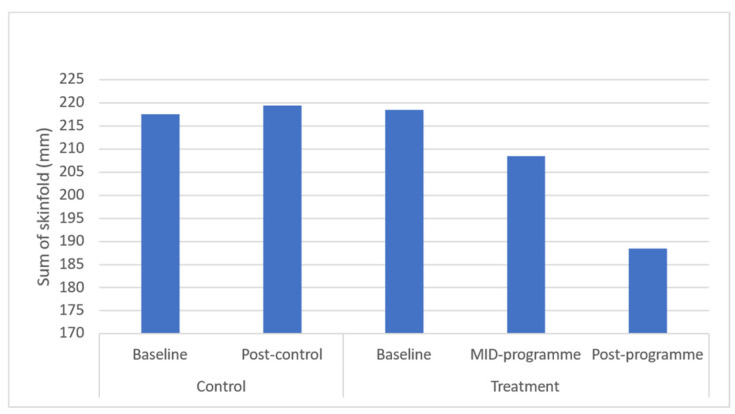
Effects of a community-based mind–body (Tae-Bo) physical exercise programme on sum of skinfolds in rural black overweight and obese women with manifest risk factors for multimorbidity. Legend: mm: millimetres: kilograms.

**Figure 6:**
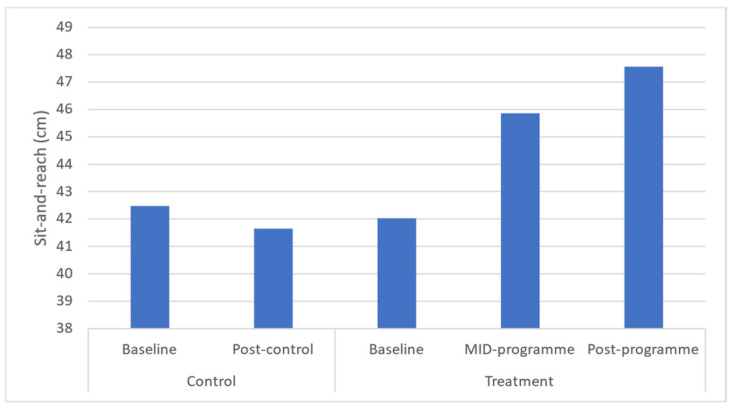
Effects of a community-based mind–body (Tae-Bo) physical exercise programme on sit-and-reach in rural black overweight and obese women with manifest risk factors for multimorbidity. Legend: cm: centimetres.

Results demonstrated significant (*p* ≤ 0.05) improvements in body weight (*p* = 0.043), BMI (*p* = 0.037), and waist (*p* = 0.031) and hip circumferences (*p* = 0.040) across the entire programme (Figure 1, Figure 2, Figure 3, Figure 4 and Figure 5). However, no significant change was found in sum of skinfolds following the programme (*p* = 0.057), despite a medium effect size being found (Figure 6). For body composition, moderate effect sizes were found for all the remaining body composition variables at completion of the programme. When evaluating the time-course changes in body composition, BMI was found to initially remain stable at mid-intervention followed by a decrease. Hip circumference was found to decrease by mid-programme but plateaued for the remainder of the programme.

**Figure 7:**
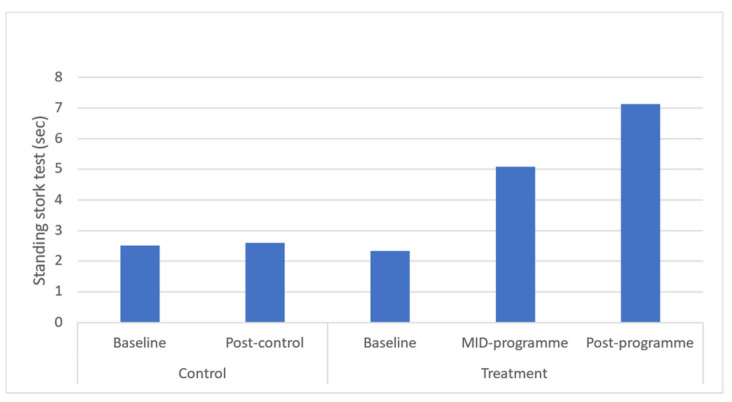
Effects of a community-based mind–body (Tae-Bo) physical exercise programme on standing stork balance in rural black overweight and obese women with manifest risk factors for multimorbidity. Legend: sec: seconds.

Flexibility was found to be significantly increased at both mid- and post-programme (*p* = 0.033 and *p* = 0.025, respectively) (Figure 7). A medium effect size was found for flexibility across the programme. Time-course changes revealed that these increases occurred across the entire programme.

**Figure 8:**
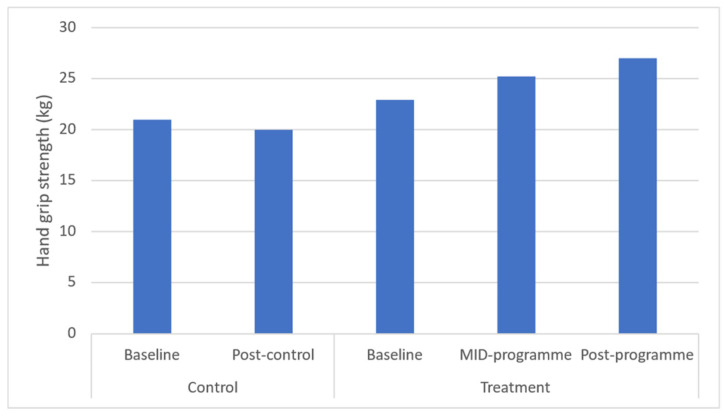
Effects of a community-based mind–body (Tae-Bo) physical exercise programme on hand grip strength in rural black overweight and obese women with manifest risk factors for multimorbidity. Legend: kg: kilograms.

Static balance was found to be significantly increased at both mid- (*p* = 0.022), and post-programme (*p* = 0.019) (Figure 8). Following the programme, a large effect size was found for balance. Time-course changes demonstrated that these increases occurred across the entire programme.

**Figure 9:**
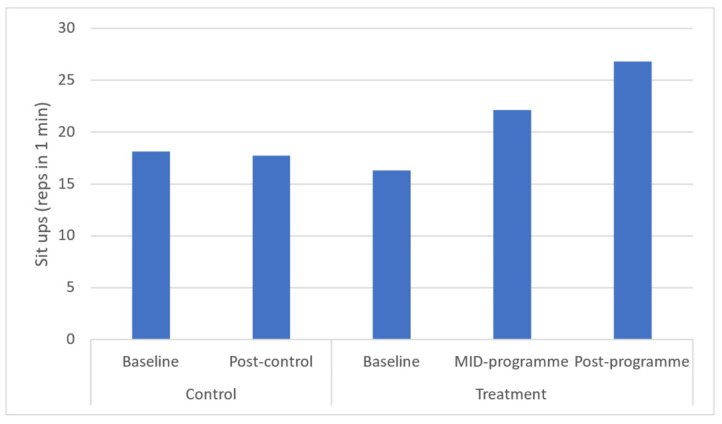
Effects of a community-based mind–body (Tae-Bo) physical exercise programme on sit ups in rural black overweight and obese women with manifest risk factors for multimorbidity. Legend: min: minute.

Hand grip strength was found to be significantly increased at both mid- (*p* = 0.034), and post-programme (*p* = 0.029), with a large effect being found for this health-related physical fitness parameter (Figure 9). Time-course changes reveal that these increases occurred across the entire programme. Further, sit-up performance was found to be increased at both mid- and post-programme (*p* = 0.021 and *p* = 0.018, respectively). Time-course changes showed that these increases occurred across the entire programme.

**Figure 10:**
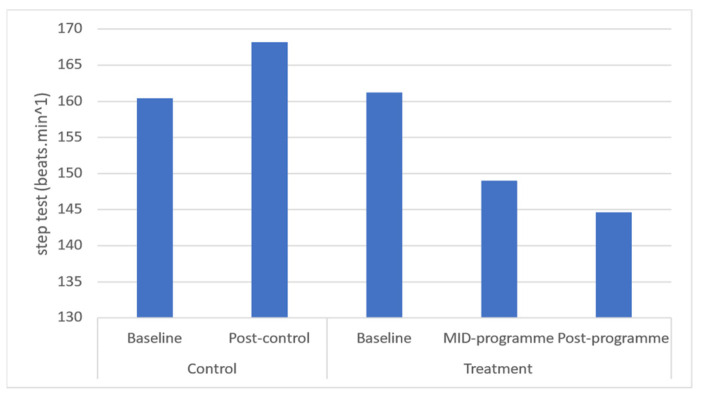
Effects of a community-based mind–body (Tae-Bo) physical exercise programme on step test performance in rural black overweight and obese women with manifest risk factors for multimorbidity. Legend: beats.min^−1^: beats per minute.

Cardiorespiratory endurance was found to be significantly increased from baseline to mid-programme (*p* = 0.017) and from baseline to post-programme (*p* = 0.011) (Figure 10). Following the programme, a large effect size was found for cardiorespiratory endurance. Time-course changes revealed that these increases occurred across the entire programme.

When compared to the multiple baseline period, significant (*p* ≤ 0.05) differences were found for all health-related physical fitness parameters following the 10-week intervention programme. These results indicate that the community-based mind–body physical activity programme had a significant and simultaneous effect on health-related physical fitness.

Rank-ordering revealed that the largest favourable change occurred in cardiorespiratory endurance, followed by sum of skinfolds, muscle endurance, BMI, waist circumference, body weight, flexibility, balance, hand grip strength, and hip circumference. Further, BMI was negatively associated with sit-and-reach (−0.372; *p* = 0.041), standing stork performance (−388, *p* = 0.042), hand grip strength (−0.408; *p* = 0.034), sit-up performance (−0.391; *p* = 0.032), and cardiorespiratory fitness (−0.432; *p* = 0.044), while being positively associated with body weight (0.675; *p* = 0.025), waist circumference (0.733; *p* = 0.019), hip circumference (0.425; *p* = 0.026), and sum of skinfolds (0.398; *p* = 0.039). Cardiorespiratory fitness was negatively associated with body weight (−0.527; *p* = 0.032), BMI (−0.432; *p* = 0.044), waist (−0.642; *p* = 0.036) and hip circumference (−0.436; *p* = 0.047), and sum of skinfolds (−0.342; *p* = 0.049), while being positively associated with sit-and-reach (0.512; *p* = 0.030), standing stork performance (0.776 *p* = 0.025), hand grip strength (0.534; *p* = 0.035), and sit-up performance (0.705; *p* = 0.027).

Recording of the training intensity at each session revealed that self-selected training intensity, within the given ranges, steadily increased weekly, rising from an RPE of 11.26 ± 1.28 in week 1 to an RPE of 15.35 ± 1.33 in week 10 (Table 2). This increase indicates a 27% increase in training intensity across the programme.

## 4. Discussion

This programme aimed to investigate if a community-based mind–body physical activity programme could be implemented in a low-resource setting and if such a programme results in positive outcomes in health-related physical fitness in rural black overweight or obese women with manifest risk factors for multimorbidity.

This programme demonstrated an 89.6% adherence rate for this programme with 60 of the initial 67 participants completing the programme, a 96.7% compliance when considering the overall required sessions, and recorded no adverse events associated with the programme. The main findings of this study showed that provision of a community-based mind–body physical activity programme can simultaneously improve several health-related physical fitness in rural black overweight or obese women with manifest risk factors. This is similar to a 6-month (~24-week), thrice-weekly, 90 min multimodal (aerobic, strength, balance, and flexibility training) community-based intervention taking place at a community-based fitness centre in Toronto, Canada, where participants attended a median of 18/25 (72%) weekly supervised sessions [33].

In this programme, body weight significantly decreased which was similar to that following a 6-month community-based physical activity and nutritional guidance programme in 152 rural and urban Japanese women aged 40–74 years [34], which observed a decrease from 52.7 to 51.9 kg in body weight. Further, the present programme found a decrease in BMI from 32.6 to 30.60 kg·m^−2^ after 10 weeks. In turn, the 6-month community-based physical activity and nutritional guidance programme failed to improve BMI in the 152 rural and urban Japanese women aged 40–74 years [34]. These decreases in BMI observed in the present study from the upper limit of class I (30–34.9 kg·m^−2^) mean classification of obesity to the lower limit of class I are noteworthy in that research has shown that BMI is strongly correlated with measurements of body fat [35]. In addition, BMI presents a simplistic method for community health workers to screen those community-dwellers that might be at greater risk of all-cause morbidity and mortality. Further, in this programme, waist circumference decreased from 87.49 to 81.48 cm, demonstrating a 6.6% improvement in this outcome. This improvement is significant in that the 10-week programme reduced mean waist circumference away from the recommended cut-off point by the National Heart Lung and Blood Institute (NHLBI) of 88 cm for women [36]. This improvement is clinically relevant in that this proxy measurement is essential for predicting morbidity and risk of death [37] due to it being closely associated with measures of visceral fat [11]. The findings of this improvement are supported by a decrease in waist circumference (from 82.4 to 79.9 cm or 3.0%) following the 6-month community-based exercise and nutritional guidance programme in 152 rural and urban Japanese women aged 40–74 years [34]. Similarly, a 12-week, twice-weekly community-based physical activity intervention using concurrent resistance training and high-intensity interval training (HIIT) (*n* = 12), and concurrent resistance training and moderate-intensity continuous training (MICT), resulted in average reductions in waist circumference of 3 to 4 cm in both groups [38]. While the present programme failed to elicit improvements in sum of skinfolds, the programme of Uritani et al. [34] demonstrated a decrease in body fat percentage following their six-month community-based exercise and nutritional guidance programme.

This programme significantly improved sit-and-reach flexibility which is similar to that following a 12-week, twice-weekly community-based physical activity intervention using concurrent resistance training and high-intensity interval training (HIIT) in low-income older women (aged > 65 years) [38]. That programme resulted in an average increase of ~15.8% compared to this study’s 5.6% increase. Possible explanations for that enhanced increase in sit-and-reach performance could be related to the age of the sample utilised and the high intensity of that programme when compared to the present study. Further, following a 6-month, thrice-weekly, 90 min multimodal (aerobic, strength, balance, and flexibility training) programme, middle-aged participants (51 years) observed a median 1.74 cm increase in sit-and-reach performance [33]. It is worth noting that the improvements in this programme arose despite the mean values found in this programme at baseline being above those considered excellent (≥29 cm) for this sample aged 25 ± 5 years [18]. Adequate range of motion is clinically important, especially in overweight or obese individuals, to reduce their risk of injuries and falls, improve muscle blood flow, enable muscles to work most effectively, and improve their ability to do daily activities [39].

Balance, as assessed via the standing stork test, was improved following this community-based mind–body physical activity programme in rural black overweight or obese women. Although a previous study utilising a 12-week, twice-weekly, 50 min concurrent aerobic and resistance training programme in urban facilities within a community demonstrated that the programme improved balance in multiple conditions, it was found that the programme did not improve balance in a static condition on a level surface [40]. The difference observed when compared to the present programme may be related to the difference in the population sampled as that programme utilised 107 individuals 65 years of age or older, whereas the current study utilised 60 young rural black adult females aged 25 ± 5 years. This because the underlying mechanisms affecting balance in these two populations are different, with the elderly experiencing balance problems because of a variety of issues including, inter alia, sudden changes in blood pressure, neurological conditions, lack of circulation, and medications. On the other hand, balance challenges in overweight or obese individuals relate to weight not always being carried or distributed evenly throughout their body and lack of physical fitness. The improvement in stork standing duration following this programme from a mean of 2.34 to 7.13 s demonstrates an increase in normative level from poor (<3 s) to below average (3–7 s) [41]. These improvements in balance are notable in that balance is important for many activities of daily living (ADLs) and preventing falls and other injuries, especially in overweight or obese individuals, since their weight is not always carried or distributed evenly throughout their body [42].

This 10-week programme resulted in an approximate 4 kg increase in hand grip strength from 22.9 to 27.0 kg in this sample of rural black overweight or obese women with manifest risk factors. This increase is supported, albeit on a smaller scale, by a previous study utilising a 12-week, twice-weekly community-based physical activity intervention using concurrent resistance training and high-intensity interval training (HIIT) in low-income older women (aged >65 years) [38]. In that study, researchers found an average increase in hand grip of 15.8%. These increases in hand grip strength are important since hand grip strength is often used as an indicator of overall muscle strength and low hand grip strength is linked to a variety of poor health outcomes, including functional disabilities, chronic morbidities, and all-cause mortality [43]. It is for this reason that many public health initiatives and programmes target the preservation of muscle strength [43]. It must be noted that the mean values observed in this study are considered low at baseline and at post-programme (ACSM), indicating that further manipulation of programme design variables (i.e., volume, frequency, intensity, type, and/or duration) may be required to optimise hand grip strength improvements.

Further, this programme demonstrated increases in the maximum number of sit-up repetitions achieved in one minute. Specifically, the present programme increased repetitions from 16.3 to 26.8. This improvement effectively resulted in the mean values increasing from a low value to a satisfactory value for this sample [19]. Similarly, after following a 6-month, thrice-weekly, 90-min multimodal (aerobic, strength, balance, and flexibility training) programme, middle-aged participants were able to complete a mean additional 2.89 sit ups [33]. Although lower than the mean observed in the present programme, those findings are consistent with improvements in muscular endurance following a community-based intervention. These findings are important since muscular endurance is essential for the maintenance of good posture and stability for long periods which are required to carry out functional activities of daily living (ADLs). A lack of muscular endurance results in muscular fatigue which is also a major cause of muscle strains and bone fractures [44], signifying the importance of optimising muscular endurance, especially in women.

The improvement in cardiorespiratory fitness following this programme is critical. This is because cardiorespiratory fitness is related to functional capacity and has been shown to be a strong and independent predictor of all-cause and disease-specific mortality. This association is so strong that cardiorespiratory fitness is considered one of the key predictors of longevity [45]. The present programme’s improvements in cardiorespiratory are supported by those of O’Brien et al. [33] whose 6-month, thrice-weekly, 90 min multimodal (aerobic, strength, balance, and flexibility training) programme using middle-aged participants was found to have increased their mean peak oxygen consumption (VO_2peak_) by a median of 0.56 mL.kg^−1^.min^−1^. The YMCA step test, as utilised by this programme, does not utilise oxygen consumption (VO_2_) or metabolic equivalents (METs) like other tests but rather the resting heart rate after a 60 s rest [46]. In this regard, although there was an improvement in resting heart rate from 161 to 145 beats.min^−1^, and this measure of physical fitness observed the largest change out of all measures, both baseline and post-programme cardiorespiratory fitness can still be regarded as “very poor” [46].

Results of the self-selected training intensity revealed that mean RPE increased from 11.26 in week 1 to 15.53 in week 10. This indicates a shift from a mean moderate intensity (12 to 14) to a mean vigorous training intensity (15 to 17) [19]. This increase in training intensity is important for several reasons in that these increases demonstrate not only a continuous adaptation to the programme but also a shift to a more vigorous level of training that could potentially lead to enhanced improvements in health-related physical fitness.

In summary, while improvements in health-related physical fitness have been previously demonstrated in overweight or obese women using a variety of physical activity interventions, these studies are individualised laboratory- and/or home-based studies and are usually performed in high-income countries (HICs) where the ecological validity and practicality of such programmes are limited [47]. In this regard, the present study also utilised a quasi-experimental design, because when compared to randomised controlled trials (RCTs), such studies have shown to have higher internal validity (i.e., better establishment of the causal relationship between the intervention and the outcome) and higher external validity (i.e., the findings can be better generalised to other contexts and settings). In addition, as part of the approval process of this study, tribal leaders indicated that services could not be denied to some participants, as in RCTs. Where possible to compare in a community setting, the rate of improvements in health-related physical fitness in this programme following a community-based mind–body physical activity programme in rural black overweight or obese women with manifest risk factors in a real-life low-resource scenario was similar to those of other community-based physical activity interventions using rural and urban middle-aged and aged women. Following the successful outcomes of this programme in a rural community with manifest NCD risk, these findings could be used to inform the planning of future RCTs and/or implementation science interventions in this context. Although many experimental and meta-analytical studies have previously demonstrated the effects of physical activity and diet, either alone or in combination on body composition [9], there are no direct links between diet and other health-related physical fitness parameters, such as cardiorespiratory endurance, muscular endurance, muscular strength, body composition, flexibility, and balance. In addition, many physical activity interventions take place in clinical and not community settings and in well-resourced settings, communities, or countries. As such, to our knowledge this is the first trial that examined and demonstrated the effectiveness of a community-based mind–body physical activity programme in a rural community in an LMIC.

The strengths and limitations of the study must be acknowledged. This quasi-experimental study was strengthened using an in situ physical activity programme within the community itself. It also made use of a large sample when compared to previous studies and made use of cost-effective objectively measured outcomes as would be utilised in that low-resource setting, increasing its ecological validity or generalisability to other low-resource settings, such as in LMICs. This study also had some limitations. Firstly, results of this study may not be generalisable to male populations and further research in this area should make use of both male and female groups, where culturally plausible. This study also made use of a single rural setting and may not represent other rural centres. Problematically, since this is, to our knowledge, the first trial that examined and demonstrated the effectiveness of a community-based physical activity programme in a rural community in an LMIC, especially targeted at women, it appears that research to support evidence-based practice is focused primarily on the elderly. While physical activity interventions combined with dietary intervention may result in enhanced improvements in body composition, community dieticians are unwilling to practice in rural areas due to a plethora of reasons including a lack of career opportunities, the recruitment of students into health professions from incongruous backgrounds, inappropriate training, bureaucratic problems, poorly equipped hospitals, and social isolation [48].

Further, when evidence of such intervention programmes is forthcoming, they are performed in “community-dwelling” individuals at laboratories or in home settings. This focus then shifts research and public health initiatives to the secondary and tertiary prevention of NCDs rather than the primary prevention level. As such, the findings of this programme could inform the planning of future RCTs and/or implementation science interventions in rural and/or low-resource community settings.

Future studies should make use of larger sample sizes that are representative of the specific communities to ensure assessment of the adequacy of instrumentation or providing better statistical estimates. Further, longer studies need to be employed to determine inter alia scalability and long-term behaviour change following such interventions and if any maintenance or continued improvements forthcoming from such interventions occur. Future studies should also include attempts to educate participants on how to estimate spontaneous physical activity at home using accelerometers and questionnaires and evaluate individual risk with international (i.e., European) scores of cardiovascular risk. In addition, future studies should attempt to determine if improvements in health-related physical fitness occur with simultaneous improvements in other emerging risk factors (i.e., homocysteine, fibrinogen, and C-reactive protein) [49,50]. When combined with this intervention’s proven ability to simultaneously improve multiple health-related physical fitness parameters in this sample of rural black overweight and obese women with manifest risk factors for multimorbidity, the low cost of implementation and low reliance on agency may present this as a potential feasible intervention in other similar rural black South African communities. However, specific feasibility or implementation science research needs to be conducted to support its feasibility.

## 5. Conclusions

Results of this trial evaluate a community-based mind–body physical activity programme on health-related physical fitness in rural black overweight or obese women with manifest risk factors in a real-life low-resource scenario. Specifically, this 10-week community-based mind–body (Tae-Bo) PA programme improved all health-related physical measures, barring sum of skinfolds. This approach, with its rigorous methods and standardised operating procedures for the conduction of the intervention, will allow valid conclusions for the implementation of community-based mind–body physical activity programmes in community-dwelling individuals with multimorbidity. In addition to informing the planning of future RCTs and/or implementation science interventions in this context, this programme’s finding could serve as an opportunity for translation into routine practice in rural settings in LMICs. Such a community-based mind–body programme presents an opportunity to level health inequalities and positively improve health-related physical fitness in low-resource communities irrespective of the underlying barriers to participation.

## Figures and Tables

**Table 1:** Effects of a community-based mind–body (Tae-Bo) physical exercise programme on health-related physical fitness in rural black overweight and obese women with manifest risk factors for multimorbidity.

	Control Group (*n* = 65)				Treatment Group (*n* = 60)			
	Baseline (*n* = 60)	Post-Control (*n* = 60)	Baseline (*n* = 60)	Mid-Programme (*n* = 60)	Post-Programme (*n* = 60)	Δ% Baseline to Mid	Δ% Baseline to Post	Effect Size
Body weight (kg)	80.73 ± 16.61 (77.50–87.28)	80.53 ± 12.43 (76.51–87.29)	81.02 ± 15.08 (77.93–86.10)	79.08 ± 15.07 * (75.53–84.62)	77.13 ± 15.92 * (72.64–81.63)	↓2.5	↓5.9	0.3
BMI (kg·m^−2^)	33.4 ± 6.02 (30.43–33.71)	33.1 ± 6.22 (30.54–33.06)	32.6 ± 5.84 (30.61–33.59)	32.3 ± 3.14 * (31.49–32.11)	30.60 ± 5.16 * (28.78–31.42)	↓0.9	↓6.7	0.2
Waist circum (cm)	86.08 ± 11.25 (86.01–91.77)	87.20 ± 10.57 (85.46–91.64)	87.49 ± 9.65 (85.19–90.60)	83.57 ± 9.24 * (81.47–86.66)	81.48 ± 10.71 * (79.52–84.44)	↓4.5	↓6.6	0.6
Hip circum (cm)	117.89 ± 10.53 (117.25–128.65)	118.91 ± 8.99 (116.87–127.58)	118.49 ± 9.65 (115.19–121.60)	116.49 ± 9.65 (116.19–119.60)	116.49 ± 10.65 (113.19–119.60)	↓2.0	↓3.5	0.4
Sum of skinfolds (mm)	217.55 ± 28.67 (203.87–226.43)	219.47 ± 30.05 (202.92–224.11)	218.49 ± 33.65 (205.19–221.60)	208.49 ± 34.65 (195.19–211.60)	188.49 ± 34.65 (175.19–191.60)	↓5.72	↓11.7	0.7
Sit-and-reach (cm)	42.48 ± 9.88 (41.43–47.42)	41.65 ± 10.01 (40.73–46.70)	42.02 ± 8.08 (39.93–44.10)	45.87 ± 7.56 * (43.91–47.82)	47.57 ± 7.5 * (45.63–49.5)	↑3.85	↑5.6	−0.7
Standing stork test (s)	2.51 ±1.64 (1.77–2.43)	2.60 ±1.43 (1.75–2.68)	2.34 ±1.58 (1.93–2.76)	5.08 ±2.07 * (4.53–5.62)	7.13 ± 1.92 * (6.64–7.63)	↑2.7	↑4.8	−2.7
Hand grip strength (kg)	20.98 ± 3.87 (22.65–24.54)	19.98 ± 5.46 (21.41–24.17)	22.91 ± 4.39 (21.76–24.07)	25.22 ± 4.55 * (24.03–26.42)	27.00 ± 3.96 * (25.96–28.04)	↑2.3	↑4.1	−0.9
Sit ups (reps in 1 min)	18.14 ± 8.66 (14.02–18.43)	17.75 ± 7.31 (14.33–18.28)	16.32 ± 6.32 (14.68–17.95)	22.12 ± 6.13 * (20.53–23.7)	26.83 ± 6.13 * (25.48–28.19)	↑5.8	↑10.5	−1.8
Step test (beats.min^−1^)	160.4 ± 27.12 (152.5–164.4)	168.2 ± 31.03 (151.7–176.1)	161.20 ± 20.81 (155.8–166.6)	149.00 ±19.92 * (143.8–154.2)	144.60 ±16.92 * (140.1–149.0)	↓12.2	↓16.6	0.9

Values are means ± SD and (confidence interval); * *p* ≤ 0.05 compared to baseline; Δ%: percentage change; kg: kilograms; BMI: body mass index; kg·m^−2^: kilograms per square metre; cm: centimetres; circum: circumference; mm: millimetres; sec; seconds; reps: repetitions; beats.min^−1^: beats per minute.

**Table 2:** Self-selected training intensity at each physical activity session.

Week	Training Intensity in Rating of Perceived Exertion (RPE)
1	11.26 ± 1.28
2	12.46 ± 1.32
3	12.07 ± 1.33
4	12.44 ± 1.22
5	13.49 ± 0.99
6	13.81 ± 0.98
7	13.95 ± 1.47
8	14.75 ± 1.42
9	14.86 ± 1.41
10	15.35 ± 1.33

Values are means ± SD.

## Data Availability

The data that support the findings of this study are available from the corresponding author upon reasonable request.
